# Evaluation of efficacy and safety of glycopyrrolate - xylazine - propofol anesthesia in buffalo calves

**DOI:** 10.14202/vetworld.2015.251-256

**Published:** 2015-03-04

**Authors:** Sandeep Potliya, Ashok Kumar, Sandeep Kumar, Sukhbir Singh, Sarvan Kumar

**Affiliations:** 1 Department of Veterinary Surgery and Radiology, College of Veterinary Science, Lala Lajpat Rai University of Veterinary and Animal Sciences, Hisar, Haryana, India; 2 Department of Veterinary Physiology and Biochemistry, College of Veterinary Science, Lala Lajpat Rai University of Veterinary and Animal Sciences, Hisar, Haryana, India; 3 Department of Veterinary Pathology, College of Veterinary Science, Lala Lajpat Rai University of Veterinary and Animal Sciences, Hisar, Haryana, India

**Keywords:** buffalo calves, propofol, xylazine, glycopyrrolate

## Abstract

**Aim:**

To evaluate the efficacy and safety of glycopyrrolate - xylazine - propofol anesthesia in buffalo calves.

**Materials and Methods:**

The study was conducted on six clinically healthy male buffalo calves, 6-12 months of age, and weighing between 130 and 170 kg. In all the animals; glycopyrrolate (0.01 mg/kg, IM), xylazine (0.1 mg/kg, IM) and 1% propofol as single bolus (1.5 mg/kg, intravenous), were administered. The parameters observed included behavioral changes, physiological; hematological and blood biochemical parameters.

**Results:**

Muzzle and nostrils became dry in all the animals after glycopyrrolate administration. A decrease in spontaneous activity and mild cutaneous analgesia was noticed after xylazine administration. After administration of propofol, loss of swallowing reflex, palpebral reflex, corneal reflexes, periosteal reflex and complete analgesia was observed. There was no significant change in rectal temperature and heart rate. However, heart rate remained elevated during anesthesia. Respiratory rate decreased significantly after propofol administration. There was a significant increase in plasma glucose after the xylazine and propofol administration which remained elevated till recovery. A significant decrease in chloride level was seen after propofol administration.

**Conclusions:**

Glycopyrrolate - xylazine - propofol anesthetic combination may safely be used for short duration anesthesia in buffalo calves.

## Introduction

General anesthesia is a state of reversible unconsciousness produced by a process of controlled, drug-induced intoxication of the central nervous system (CNS) in which the patient neither perceives nor recalls noxious stimuli [1]. No single anesthetic drug produces all of the components of general anesthesia without depressing some vital organ function. So a multiple drug approach (balanced anesthesia) is exploited to diminish sensory, motor, sympathetic and parasympathetic reflex activities, and to attenuate individual components of the anaesthetic state.

Anesthesia with rapid, smooth induction and lesser recumbency time are desirable in large ruminants. Propofol (2, 6-diisopropylphenol) is an intravenous anaesthetic agent characterized by rapid onset and fast recovery without any cumulative effect [2,3]. Propofol has advantage over thiopentone and ketamine in that elimination is more rapid, and recovery is quick [4]. Propofol was found a better induction agent and a safe alternative to thiopentone sodium for anesthesia induction in water buffaloes [5]. Propofol was found to be a safe injectable anaesthetic agent for induction of general anesthesia in buffalo calves, but transient apnoea was observed to be the most prevalent side effect [6,7]. Various pre-anesthetic combinations have been used with propofol; to prolong the duration of anesthesia along with shorter recovery time, thereby improving quality of anesthesia [8-11]. Xylazine is an alpha2-adrenergic receptor agonist with potent sedative, analgesic and muscle relaxant activity and was widely used in large animals for restraining [12]. Since α_2_-agonists produces dose-dependent sedation and ruminants assumes lateral recumbency when only moderated sedated [1], a lower dose of xylazine may be used for premedication. Xylazine was found a better anaesthetic before propofol administration than midazolam in dogs due to an early onset, longer duration of analgesia and minimum adverse effects [8]. Propofol along with xylazine produced better quality of anesthesia and recovery than xylazine- ketamine combination in equine gelding [13] and was useful along with xylazine and acepromazine in anesthetic procedures requiring longer duration in field ambulatory equine practice [14]. Glycopyrrolate, a synthetic quaternary ammonium antimuscarinic agent, has received attention for anesthetics use in veterinary medicine [15] and have been used in buffalo calves to offset the side effects on hemodynamic parameters induced by xylazine [16].

But a comprehensive and planned study on the effects of glycopyrrolate - xylazine - propofol anesthesia has not been done in buffaloes; therefore the present study was under taken with the objective to evaluate efficacy and safety of glycopyrrolate - xylazine - propofol anesthesia in buffalo calves.

## Materials and Methods

### Ethical approval

The study was undertaken after taking necessary approvals from the Institutional Animal Ethical Committee of the University.

### Animals

The investigation was conducted on six clinically healthy male murrah buffalo calves (*Bubalus bubalis*), 6-12 months of age, and weighing 130-170 kg. The feed and water were withheld for 12 h. Anesthetic protocol included administration of glycopyrrolate (0.01 mg/kg, IM); 15 min later xylazine (0.1 mg/kg, IM) and then at 15 min of xylazine, induction of anesthesia was done with single bolus of 1% propofol (1.5 mg/kg, IV). Following parameters were included in the study.

### Behavioral changes

The animals were observed to record the behavioral changes, namely: Spontaneous motor activity, lowering of head, onset of salivation, urination, defaecation, weak time (time elapsed from administration of drug to onset of ataxia), down time (time elapsed from administration of drug to onset of sternal or lateral recumbency) and muscle relaxation. Depth of anesthesia was judged by observing swallowing reflex, palpebral reflex, corneal reflex and closing of the eye. Analgesic effect of drugs were judged by observing physical response of the animal to cutaneous hypodermic needle pricks on fetlock, base of tail, abdomen, base of horn and scratching of rib periosteum with needle after its subcutaneous insertion. Recovery from anesthesia was taken to have occurred on return to sternal recumbency, regaining of head rightening reflex, standing time with ataxia, browsing time (time elapsed from administration of drug to occasional nibbling of grass) and complete recovery without ataxia.

### Physiological parameters

Rectal temperature, heart rate and respiratory rate along with the ambient temperature were recorded just before administration of the drug(s), to form the base values. These parameters were investigated at 15 min of glycopyrrolate, at 15 min of xylazine, at 5 min of propofol, at recovery from anesthesia and at 24 h of glycopyrrolate administration.

### Hematological studies

Blood samples; collected by jugular venipuncture, before administration of the drug(s), at 15 min of xylazine, at 5 min of propofol, at recovery and at 24 h of glycopyrrolate administration; were used for hematological studies which included hemoglobin (Hb) and packed cell volume (PCV). Hb was estimated by Sahli’s haemoglobinometer and PCV by Wintrobe’s tube method [17].

### Blood-biochemical studies

Blood samples for analysis of biochemical parameters were collected in two sets of test tubes. One set of test tube containing 3.8% sodium fluoride (@ 10 mg/mm of blood) for determining glucose and other set containing heparin (10 units/ml) for estimation of urea nitrogen, creatinine, total proteins, albumin, globulin, sodium, potassium, chloride, alanine aminotransferase (ALT), aspartate aminotransferase (AST), alkaline phosphatase (ALKP) and bilirubin. Plasma was harvested by centrifugation at 1500 rpm for 30 min. and stored at −20°C in the deep freezer. Biochemical parameters were analysed with EM 200™ (Erba Mannheim, Germany) automated random access clinical chemistry analyzer using commercially available Transasia XL system pack kits procured from M/S Transasia Biomedical Limited, Mumbai. Sodium and potassium were analysed by flame photometry method at 589 nm for sodium and 768 nm for potassium. Chloride was measured by colorimetric method by using kit by Bayer Diagnostic India Limited.

### Statistical analysis

The statistical analysis of data was done by one-way-analysis of variance and Duncan’s multiple range test [18].

## Results

### Behavioral changes

The effects of administration of glycopyrrolate-xylazine-propofol combination on behavioral parameters are shown in Table-1. Muzzle and nostrils became dry in all the animals at 19.83±2.77 min of glycopyrrolate administration. A decrease in spontaneous activity was seen in all the animals after xylazine administration. Lowering of head was observed in four animals. Onset of salivation was noticed in all the animals at 6.50±0.43 min of xylazine administration. Urination was observed in two animals. Two animals defecated after xylazine administration. Mild cutaneous analgesia was observed in all the animals on pin-pricks at abdomen, thorax, tail, base of horn. All the animals assumed lateral recumbency at 13.17±1.50 min of xylazine administration.

**Table-1:** Mean and SE in time format of minute for different behavioral characteristics related to the onset of CNS depression and recovery from CNS depression induced by administration of glycopyrrolate-xylazine-propofol combination.

Reflexes	Mean time (min)±SE
Muzzle dryness (after glycopyrrolate administration)	19.83±2.77
Weak time (after xylazine administration)	6.0±0.82
Onset of salivation (after xylazine administration)	6.5±0.43
Down time (after xylazine administration)	13.17±1.50
Loss of swallowing reflex (after propofol administration)	1.68±0.05
Limb relaxation (after propofol administration)	1.88±0.08
Loss of periosteal scratch reflex (after propofol administration)	2.25±0.18
Eyes closed (after propofol administration)	3.32±0.61
Ventral rotation of the eyeball (after propofol administration)	3.67±0.61
Ear flapping (after propofol administration)	9.50±1.44
Regain of periosteal reflex (after propofol administration)	18.17±1.80
Eyes open (after propofol administration)	20.0±1.81
Limb tone regain (after propofol administration)	29.67±1.48
Regaining of head righting reflex (after propofol administration)	32.0±1.78
Sternal recumbency (after propofol administration)	52.17±3.26
Standing time (after propofol administration)	63.0±4.99
Browsing time (after propofol administration)	105.0±7.33
Complete recovery (after propofol administration)	132.0±8.63

CNS: Central nervous system, SE: Standard error

After propofol administration, swallowing reflex abolished at 1.68±0.05 min. Palpebral and corneal reflexes were lost at 1.92±0.05 min and 2.05±0.10 min of propofol administration, respectively. Transient apnoea (30-45 s) was observed in four animals, and the animals regained respiration without any resuscitation. There was complete analgesia at the fetlock, base of tail, abdomen, ribs periosteum and base of the horn, and it remained until 18.17±1.80 min of propofol administration. All the animals showed an ear flapping in the mean time 9.50±1.43 min of propofol. Recovery was manifested by the opening of eyelids at 20.0±1.81 min with return of the head rightening reflex at 32.0±1.78 min. All the animals returned to sternal recumbency at 52.17±3.26 min and remained in milk-fever posture. All animals took one or two attempts to stand and succeeded at 63.0±4.99 min of propofol administration with hind limbs held apart with head down. Animals started nibbling of grass at 105.0±7.33 min. Complete recovery took 132.0±8.63 min of propofol administration.

### Physiological study

**Figure-1:**
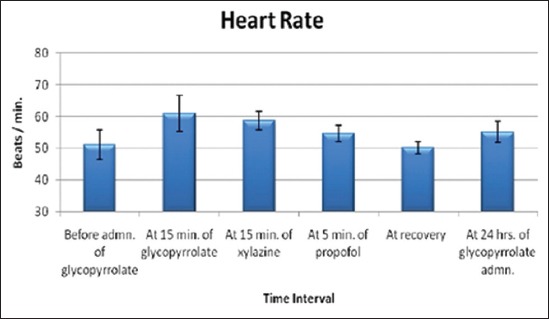
Columns showing mean values and positive-negative error lines of standard error indicating the heart rate in beats/minute (Y-axis) related to different time intervals (X-axis) of glycopyrrolate-xylazine-propofol combination administration in buffalo calves.

**Figure-2:**
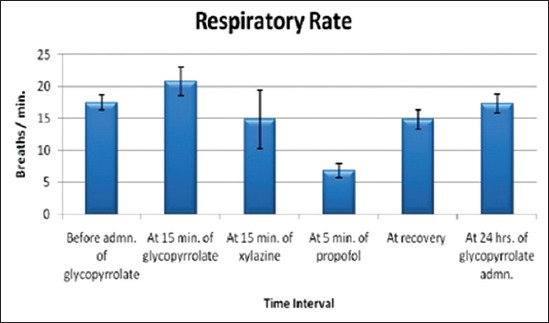
Columns showing mean values and positive-negative error lines of standard error indicating the respiratory rate in beats/minute (Y-axis) related to different time intervals (X-axis) of glycopyrrolate-xylazine-propofol combination administration in buffalo calves.

The effects of glycopyrrolate-xylazine-propofol combination on rectal temperature, heart rate (Figure-1) and respiratory rate (Figure-2) are shown in Table-2. There was no significant change in rectal temperature and heart rate; however heart rate remained higher during the anaesthetic period compared to the base value. Respiratory rate decreased significantly from the base value of 17.50±1.15 breaths/min to 6.83±1.11 breaths/min after propofol administration.

**Table-2:** Effects of glycopyrrolate-xylazine-propofol on rectal temperature, heart rate and respiratory rate in six buffalo calves.

Parameters (Units)	Before admin. of glycopyrrolate	At 15 min of glycopyrrolate	At 15 min of xylazine	At 5 min of propofol	At recovery	At 24 h of glycopyrrolate administration
Ambient temperature (°C)	25.00^b^±1.49	26.75^ab^±1.33	28.33^ab^±0.98	28.75^ab^±1.00	30.33^a^±1.38	25.92^b^±1.42
Rectal temperature (°C)	37.72^a^±0.16	37.78^a^±0.26	38.03^a^±0.25	37.90^a^±0.24	38.23^a^±0.34	37.78^a^±0.25
Heart rate (beats/min)	51.17^a^±4.62	61.00^a^±5.62	58.67^a^±3.00	54.67^a^±2.62	50.17^a^±1.92	55.17^a^±3.29
Respiratory rate (breaths/min)	17.50^a^±1.15	20.83^a^±2.20	14.83^a^±4.59	6.83^b^±1.11	14.83^a^±1.47	17.33^a^±1.50

Means with different superscripts vary significantly (p<0.05)

### Hematological study

The effects of glycopyrrolate-xylazine-propofol combination on Hb, and PCV are shown in Table-3. There were no significant variations in Hb and PCV during the entire period of observation.

**Table-3:** Effects of glycopyrrolate-xylazine-propofol on hematological parameters in six buffalo calves.

Parameters (units)	Before admn. of glycopyrrolate	At 15 min of xylazine	At 5 min of propofol	At recovery	At 24 h of glycopyrrolate administration
Hemoglobin (g/dl)	12.60^a^±0.63	12.23^a^±0.52	11.80^a^±0.55	12.20^a^±0.65	12.37^a^±0.60
PVC (%)	40.00^a^±2.08	34.50^a^±2.29	34.17^a^±2.55	35.00^a^±2.45	38.33^a^±2.56

Mean values presented here with (±) their respective SE. SE=Standard errors, PCV=Packed cell volume, Means with same superscripts do not vary significantly (p>0.05)

### Blood-biochemical study

**Figure-3:**
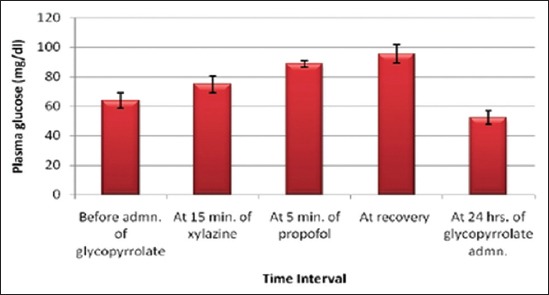
Columns showing mean values and positive-negative error lines of standard error indicating the blood glucose level in mg/dl (Y-axis) related to different time intervals (X-axis) of glycopyrrolate-xylazine-propofol combination administration in buffalo calves.

**Figure-4:**
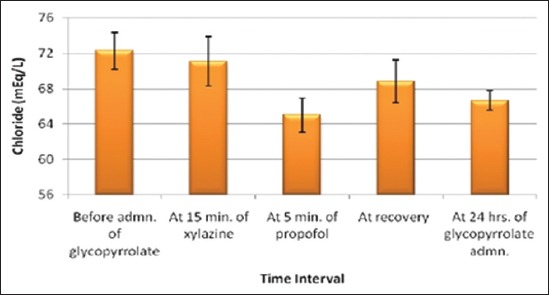
Columns showing mean values and positive-negative error lines of standard error indicating the chloride level in mEq/L (Y-axis) related to different time intervals (X-axis) of glycopyrrolate-xylazine-propofol combination administration in buffalo calves.

The effects of glycopyrrolate-xylazine-propofol combination on plasma glucose (Figure-3), urea nitrogen, creatinine, total plasma proteins, albumin, globulin, albumin: globulin ratio, chloride (Figure-4), sodium, potassium, ALT/SGPT, AST/SGOT, ALKP and bilirubin are shown in Table-4. There was a significant increase in plasma glucose (88.55±2.31 mg/dl) at 5 min of propofol and at recovery (95.47±6.52 mg/dl) which remained elevated till recovery. Significant decrease in chloride (65.02±1.94 mg/dl) was seen after propofol administration. No significant variation was seen in any other biochemical parameters.

**Table-4:** Effects of glycopyrrolate-xylazine-propofol on blood biochemical parameters in six buffalo calves.

Parameters (units)	Before admn. of glycopyrrolate	At 15 min of xylazine	At 5 min of propofol	At recovery	At 24 h of glycopyrrolate administration
Plasma glucose (mg/dl)	63.87^cd^±5.36	74.87^bc^±5.63	88.55^ab^±2.31	95.47^a^±6.52	52.47^d^±4.66
BUN (mg/dl)	9.04^a^±0.78	8.17^a^±0.82	8.08^a^±0.74	8.57^a^±0.58	8.79^a^±0.80
Creatinine (mg/dl)	1.79^a^±0.12	1.61^a^±0.09	1.51^a^±0.11	1.60^a^±0.10	1.49^a^±0.15
Total proteins (g/dl)	6.76^a^±0.23	6.45^a^±0.18	6.46^a^±0.18	6.53^a^±0.19	6.93^a^±0.22
Albumin (g/dl)	3.03^a^±0.14	2.92^a^±0.13	2.91^a^±0.12	3.04^a^±0.13	3.12^a^±0.14
Globulin (g/dl)	3.73^a^±0.14	3.52^a^±0.07	3.56^a^±0.11	3.49^a^±0.15	3.81^a^±0.16
Albumin:globulin ratio	0.82^a^±0.04	0.83^a^±0.03	0.82^a^±0.04	0.88^a^±0.05	0.82^a^±0.05
Chloride (mEq/L)	72.27^a^±2.11	71.08^ab^±2.80	65.02^b^±1.94	68.80^ab^±2.44	66.65^ab^±1.12
Sodium (mmol/L)	125.74^a^±1.58	127.01^a^±1.33	128.85^a^±1.46	129.96^a^±1.40	125.43^a^±1.44
Potassium (mmol/L)	5.43^a^±0.10	5.25^a^±0.10	5.17^a^±0.11	5.20^a^±0.07	5.42^a^±0.07
ALT/SGPT (U/L)	84.80^a^±7.07	94.17^a^±8.60	91.58^a^±7.84	74.37^a^±2.64	79.47^a^±3.18
AST/SGOT (U/L)	157.37^a^±14.96	154.57^a^±14.56	150.82^a^±13.23	160.18^a^±13.72	166.28^a^±13.20
ALKP (U/L)	89.00^a^±26.14	84.67^a^±22.35	87.33^a^±20.12	91.67^a^±25.54	79.17^a^±21.06
Bilirubin (mg/dl)	0.65^a^±0.15	0.41^a^±0.08	0.61^a^±0.17	0.71^a^±0.20	0.95^a^±0.2

Mean values presented here with (±) their respective SE. SE=Standard error, ALT=Alanine aminotransferase, AST=Aspartate aminotransferase, ALKP=Alkaline phosphatase, Means with different superscripts vary significantly (p<0.05), BUN=Blood urea nitrogen

## Discussion

### Behavioral changes

Muzzle and nostrils became dry in all the animals after glycopyrrolate administration that may occur due to decreased oral, pharyngeal, and respiratory tract secretions after glycopyrrolate administration [19]. Dryness of muzzle, mouth and nostrils has been reported in buffalo calves after glycopyrrolate administration [16]. In horses, a wide distribution of central compartment, rapid clearance, and prolonged terminal half-life have been reported following a single intravenous clinically relevant dose of glycopyrrolate [20].

A decrease in spontaneous activity, lowering of the head, lateral recumbency, and mild cutaneous analgesia occurred after xylazine administration. Good sedation along with analgesia and muscle relaxation had been reported after xylazine administration [12]. The sedative action of xylazine might be due to inhibition of lucus coeruleus neurons in pons of lower brain stem, which attenuate the sympathoadrenal responses to noxious stimuli encountered during anesthesia and surgery, and provide haemodynamic, metabolic and hormonal stability [21].

Rapid and smooth induction of anesthesia occurred after administration of a single bolus of propofol resulting in loss of swallowing reflex, palpebral reflex, corneal reflex, ventral rotation of the eye ball and complete analgesia. Rapid onset of action is caused by rapid uptake of propofol into the CNS [22] and induction of depression occur by enhancing the effect of the inhibitory neurotransmitter GABA and decreasing the brains metabolic activity [23]. Rapid and smooth induction followed by loss of swallowing reflex, palpebral reflex and the panniculus reflex have been reported in buffalo calves after a single bolus injection of propofol [6]. Transient apnoea occurred in four animals. Transient apnoea have been found to be the most prevalent side effect on induction of propofol in dogs [8] while hypotension was the most frequent adverse event that occurred after incremental or bolus dosing of propofol to cats [24]. Transient apnoea occurs in conjugation with hypercapnia, which may occur for a short duration of time following rapid bolus injection of propofol [25]. Transient apnoea after propofol administration was reported in dogs [9] and calves [6,7] while no apnoea was reported in a study in sheep [26]. Complete analgesia at the fetlock, base of tail, abdomen, ribs periosteum and base of the horn was observed in our study. Propofol has no intrinsic analgesic potency but it has been found to produce state of analgesia along with xylazine in dogs [8].

Smooth and excitement free recovery after a brief period of anesthesia occurred in the present study. Longer duration of analgesia along with prolonged recovery time was reported in dogs after premedication of propofol with xylazine [8].

### Physiological study

In our study, glycopyrrolate effectively offsets the bradycardiac effects of xylazine. Similar observations have been made after glycopyrrolate and xylazine administration in cow calves [27] and buffalo calves [16]. Heart rate remained higher during the entire period of anesthesia. Tachycardia has been observed on induction with propofol in dogs premedicated with atropine and xylazine [28].

Respiratory rate decreased significantly after propofol administration. A progressive decline in respiratory rate has been reported after the infusion of propofol alone or with xylazine-propofol infusion in dogs [8]. Respiratory rate continued to decrease significantly lower than the base line after induction of anesthesia with propofol and maintained with continuous propofol infusion in urolithic goats premedicated with dexmedetomidine [10].

### Hematological study

There were no significant variations in Hb and PCV during the entire period of observation. However, Hb and PCV showed the decreasing trends during the experiment, which might be due to sequestration of RBCs in the spleen [29]. No variations in Hb and PCV after administration of glycopyrrolate have been reported in cow calves [27] and buffalo calves [16]. Very minimal effects on the hematological parameters have been reported after xylazine administration in healthy dogs [30]. No significant variation in Hb and PCV in buffalo calves have been observed during the entire period of observation after propofol administration [6,7] while significant falls in Hb and PCV have been reported throughout the observation period in dogs during triflupromazine or diazepam premedication with propofol anesthesia [11].

### Blood biochemical study

There was an increase in plasma glucose after the xylazine and propofol administration which remained until recovery. Hyperglycaemia results from inhibition of insulin secretion. Inhibition of insulin release is mediated by α_2_-receptors in pancreatic beta cells [31]. Furthermore, xylazine may directly stimulate hepatic glucose production via α_1_-adrenoceptors in liver [19]. Moreover, during the period of anesthesia, there is decrease in basal metabolic rate of the animal and muscular activity is negligible, so utilization of glucose by muscles is also decreased probably causing slight increase in glucose concentration. However, since hyperglycaemia produced was transient in nature and within the normal physiological limit, a clinical significance cannot be attached. No significant change in glucose has been reported after glycopyrrolate-xylazine administration [16] while hyperglycaemia has been observed after propofol anesthesia in buffalo calves [6,7].

Significant decrease in chloride level was seen after propofol administration. Hypochloraemia can occur as compensating response for respiratory acidosis that can occur due to the effect of anesthetic drug on medullary respiratory centre [32]. No significant variation in chloride level has been reported in buffalo calves after administration of glycopyrrolate and xylazine [16] and after propofol administration [6,7]. No significant variations have been observed in other biochemical parameters.

## Conclusion

From a perusal of observations of the present study, it was concluded that glycopyrrolate blocked vagal mediated bradycardia caused by xylazine, and glycopyrrolate-xylazine-propofol anesthetic combination may safely be used for short duration anesthesia in buffalo calves.

## Authors’ Contributions

SP, AK and SS designed the study. SP and AK performed the study. SP, SarK and SK analysed and interpreted the Hematological and biochemical investigation. AK and SS interpreted the data. All authors participated in drafting and revision of the manuscript. All authors read and approved the final manuscript.
